# Presentation of a Rare Case of Acute Cholecystitis in the Last Trimester of Pregnancy Misdiagnosed As Acute Gastroenteritis: A Brief Review From Symptoms to Diagnosis and Effective Management of the Disease in Pregnant Women

**DOI:** 10.7759/cureus.66524

**Published:** 2024-08-09

**Authors:** Anna Thanasa, Efthymia Thanasa, Ioannis-Rafail Antoniou, Ektoras-Evangelos Gerokostas, Alexandros Leroutsos, Vasileios Papadoulis, Emmanouil M Xydias, Apostolos C Ziogas, Ioannis Thanasas

**Affiliations:** 1 Department of Health Sciences, Medical School, Aristotle University of Thessaloniki, Thessaloniki, GRC; 2 Department of Obstetrics and Gynecology, General Hospital of Trikala, Trikala, GRC; 3 Department of Anaesthesiology, General Hospital of Trikala, Trikala, GRC; 4 Department of Obstetrics and Gynecology, EmbryoClinic IVF, Thessaloniki, GRC; 5 Department of Obstetrics and Gynecology, University of Thessaly, Larissa, GRC

**Keywords:** acute cholecystitis, acute gastroenteritis, pregnancy, clinical manifestations, ultrasound, magnetic resonance imaging, conservative treatment, cholecystectomy, case report

## Abstract

This case presentation involves a 31-year-old pregnant woman (gravida 2, para 1) in her 33rd week of pregnancy, who presented to the Emergency Department of General Hospital of Trikala, in Greece, complaining of 24-hour abdominal pain, vomiting, and diarrheal stools. With a possible initial diagnosis of acute gastroenteritis, it was decided to admit the pregnant woman to the Obstetrics and Gynecology Department. Abdominal ultrasound revealed thickening of the gallbladder wall without the presence of gallstones or distension of the intrahepatic and extrahepatic bile ducts. Clinical examination by a surgical team, combined with ultrasound and laboratory findings, established the diagnosis of acute cholecystitis. After successful conservative antibiotic treatment, the patient was discharged from the department on the fifth day of hospitalization. She underwent laparoscopic cholecystectomy during the puerperal period. In this paper, after describing a case of acute cholecystitis in pregnancy, we highlight the significant diagnostic difficulties and therapeutic dilemmas regarding the management of these patients, including their reluctance to use invasive diagnostic methods and their concerns about the teratogenicity of administered drugs.

## Introduction

Gastrointestinal disorders in pregnancy can arise as a direct result of pregnancy itself (such as acid regurgitation, sialorrhea, and hyperemesis), may pre-exist (such as hepatitis), or may occur incidentally during pregnancy (such as acute appendicitis, acute cholecystitis, and acute pancreatitis). Gastrointestinal disorders requiring surgery are estimated to affect approximately 0.2% to 1% of all pregnancies [1]. The physiological changes the maternal body undergoes during pregnancy contribute to the increasing incidence of common gastrointestinal disorders in pregnant women. These disorders, including biliary tract disorders, whether or not they are acute surgical conditions, are of particular clinical interest and cause considerable concern and controversy in contemporary obstetric clinical practice [2].

Biliary tract disorders are a fairly uncommon, heterogeneous group of medical conditions that can occur during pregnancy and cause serious differential diagnostic and therapeutic problems. Pregnancy and the immediate postpartum period are associated with an increased risk of gallstone formation. The elevated serum cholesterol and lipid levels observed during a normally progressing pregnancy, combined with reduced mobility and delayed emptying of the gallbladder due to reduced wall contractility under the influence of increased levels of estrogenic hormones and progesterone, are thought to lead to the formation of biliary sludge and gallstones [3,4]. Gallstones usually remain asymptomatic, but in some cases, they may cause chronic inflammation or obstruction of the cystic duct and the development of acute cholecystitis. Asymptomatic gallstones are estimated to be found in up to 3.5% of all pregnant women. In 90% of cases, they are considered responsible for the development of acute cholecystitis during pregnancy [5,6].

In this paper, a rare case of acute cholecystitis in a pregnant woman with no history of cholelithiasis, initially misdiagnosed as acute gastroenteritis, is described. The patient was in the third trimester of pregnancy and was initially treated successfully with conservative measures. A cholecystectomy was performed in the postpartum period. The paper also highlights the significant diagnostic difficulties and treatment dilemmas in managing these patients, including their reluctance to use invasive diagnostic methods and concerns about the teratogenicity of administered drugs.

## Case presentation

**Figure 1:**
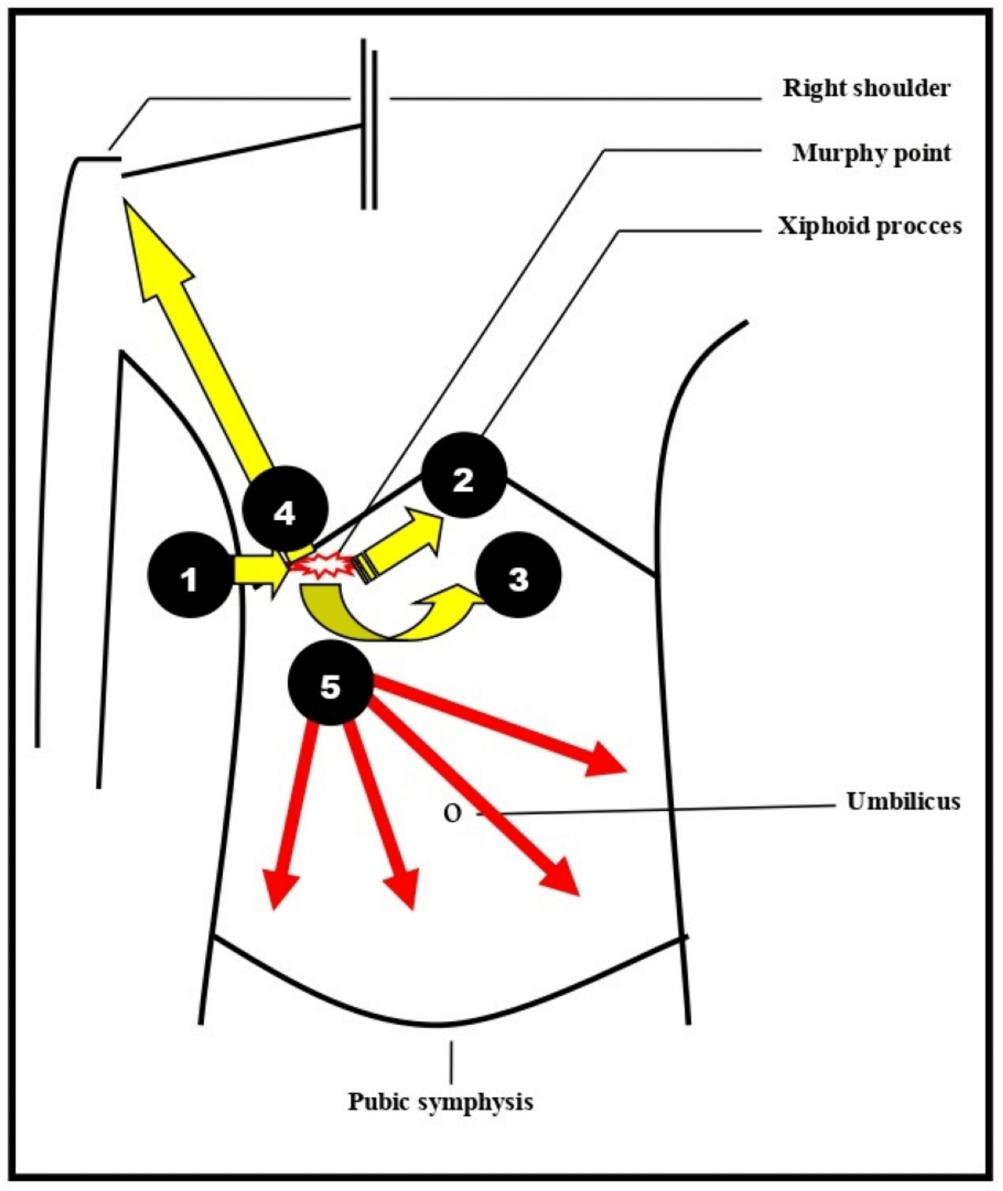
Schematic illustration of the typical and atypical locations of abdominal pain in pregnant women diagnosed with acute cholecystitis 1 - typical location of pain in the right hypochondrium (Murphy's sign positive); 2 - shift of pain from the right hypochondrium to the epigastrium; 3 - shift of pain from the right hypochondrium to the lumbar region; 4 - shift of pain from the right hypochondrium to the right shoulder; 5 - atypical location of pain diffusely in the abdomen (our case). This image was created by the authors.

A 31-year-old pregnant woman, with a history of one vaginal delivery, presented to the Emergency Department of the General Hospital of Trikala, in Greece at the 33rd gestational week, complaining of abdominal pain, vomiting, and diarrhea. The onset of symptoms had occurred approximately 24 hours earlier. The abdominal pain, which preceded the vomiting and diarrhea, was diffuse, of mild intensity, and without signs of peritoneal irritation. Her personal history was negative for pre-existing medical conditions, though she had undergone an appendectomy 15 years ago. The patient's body mass index was 26. The pregnancy had progressed uneventfully thus far. Her body temperature was 36.4°C, blood pressure was 100/70 mmHg, and pulse rate was 88 beats per minute, all within normal limits. On palpation of the abdomen, Murphy's sign, indicative of acute cholecystitis, was negative (Figure 1).

With a possible initial diagnosis of acute gastroenteritis, she was admitted to the Obstetrics and Gynecology Department. During the obstetric clinical examination, no signs of the onset of preterm labor were found. Non-stress cardiotocography and obstetric ultrasound showed no abnormal findings. Upper abdominal ultrasound revealed thickening of the gallbladder wall (>3mm) without the presence of gallstones or distension of intrahepatic and extrahepatic bile ducts. The presence of biliary sludge was evident. The pancreas, spleen, and kidneys were all free of abnormal findings. Laboratory testing on admission showed an increase in inflammatory markers and mild impairment of liver function tests (Table 1).

**Table 1:** Laboratory tests during the admission and hospitalization of the patient in the clinic Ht: Hematocrit; Hb: Hemoglobin; WBC: White Blood Cells; NEUT: Neutral; CRP: C Reactive Protein; TBIL: Total bilirubin; DBIL: Direct bilirubin; IDBIL: Indirect bilirubin; SGOT: Serum Glutamic Oxaloacetic Transaminase; SGPT: Serum Glutamate Pyruvate Transaminase; ALP: Alkaline Phosphatase; AMY: Amylase

Laboratory tests	Day of admission to the clinic	48 hours of hospitalization	7 days of hospitalization	Normal laboratory values
Ht	37.8%	34.3%	35.1%	37.7 – 49.7%
Hb	12.4 gr/dl	11.6 gr/dl	11.8 gr/dl	11.8 – 17.8 gr/dl
WBC	12.9x10^3^/ml	14.1x10^3^/ml	8.2x10^3^/ml	4 – 10.8 x10^3^/ml
NEUT	82%	86%	69%	40 – 75%
CRP	3.7 mg/dl	9.6 mg/dL	0.7 mg/dL	0.5 mg/dl
TBIL	1.45 mg/dl	1.87 mg/dl	0.91 mg/dl	0 – 1.2 mg/dl
DBIL	1.12 mg/dl	1.25 mg/dl	0.45 mg/dl	0 – 0.5 mg/dl
ΙDBIL	0.81 mg/dl	0.75 mg/dl	0.62 mg/dl	0 – 0.7 mg/dl
SGOT	57 IU/L	64 IU/L	27 IU/L	5 – 33 IU/L
SGPT	65 IU/L	71 IU/L	29 IU/L	10 – 37 IU/L
ALP	168 IU/L	151 IU/L	117 IU/L	25 – 125 IU/L
AMY	98 U/mL	87 U/mL	71 U/mL	0 – 110 U/mL

General urinalysis and stool culture were negative. Magnetic resonance imaging was not performed, as it is not feasible to perform this examination on an emergency basis in our hospital. Additionally, our patient, despite being informed about the usefulness of imaging tests in pregnancy, was reluctant to undergo any imaging diagnostic test except ultrasonography.

It was decided to maintain fluid and electrolyte balance and prescribe an appropriate diet for the diarrheal stools. An internal medicine consultation ruled out acute gastroenteritis. Clinical examination by a surgical team, combined with ultrasound and laboratory findings, reinforced the suspected diagnosis of acute cholecystitis. The patient was closely monitored and treated with antibiotics. Intravenous cefoxitin (Mefoxil) at a dose of 2 grams every eight hours and metronidazole (Flagyl) at a dose of 500 mg every eight hours for five days were administered. On the seventh day of hospitalization, the patient was discharged asymptomatic, with negative inflammatory markers and normal liver function tests. Until labor (vaginal delivery after spontaneous onset at 39 gestational weeks without complications), she did not experience any further episodes of acute cholecystitis. The patient underwent laparoscopic cholecystectomy at the end of the puerperal period.

## Discussion

Acute cholecystitis is the second most common non-obstetric cause of exploratory laparotomy in pregnancy, second only to acute appendicitis [7]. Although pregnancy predisposes to the formation of biliary sludge and gallstones, cholecystitis does not occur more frequently in pregnant compared to non-pregnant women [8]. Acute cholecystitis is estimated to affect 0.1%-0.6% of all pregnancies. It is more common in the second and third trimesters of pregnancy, while it occurs rarely in the first trimester and in the postpartum period [7]. Additionally, it is estimated that the condition is more common in pregnant women of advanced age, obese pregnant women, and multiparous women [9]. Our patient had no risk factors. She was under 35 years of age, was not obese (body mass index: 26), and this was her second pregnancy.

The clinical diagnosis of acute cholecystitis in pregnancy is challenging. The anatomical and physiological changes that occur in a normally progressing pregnancy can cause considerable difficulties in interpreting signs, symptoms, and biochemical diagnostic markers, making the clinical and laboratory assessment of the pregnant woman inevitably confusing and difficult [10]. The clinical manifestations of acute cholecystitis in pregnant women are almost identical to those in non-pregnant women. Indigestion and intolerance of fatty foods are symptoms observed in almost all cases. Nausea and vomiting are common and associated with abdominal pain [11]. Fever (above 38℃) may occur in about 30% of cases [12]. Abdominal pain is the predominant symptom. The pain is usually of acute onset, high intensity, and mainly located in the right upper quadrant of the abdomen or in the epigastrium (Figure 1). In some cases, the pain may extend to the back, right upper hemithorax, and right shoulder [9,13]. The characteristic clinical Murphy's sign, which is positive in most cases of non-pregnant patients, is not a reliable diagnostic criterion for pregnant women, especially in the last weeks of pregnancy [5]. In our patient, the atypical manifestation of abdominal pain (mild, deep, and diffuse pain in the abdomen) combined with the presence of diarrhoeal stools created significant clinical diagnostic difficulties and led to an initial misdiagnosis of acute gastroenteritis.

Similarly, laboratory tests, including inflammatory markers (white blood cell count, C-reactive protein) and liver function tests (bilirubin, transaminases, alkaline phosphatase), are not specific for the diagnosis of acute cholecystitis in pregnant women. Leukocytosis should be evaluated cautiously, since it can be observed in normal pregnancy, especially in the late stages, reaching values as high as 20,000/mL. C-reactive protein is indicative of bacterial infection in cases where its value is above 40 IU/L [14]. Elevated levels of transaminases and mild elevation of alkaline phosphatase and bilirubin levels are frequent laboratory findings early in the disease [5]. Serum alkaline phosphatase is less useful in the diagnosis of acute cholecystitis in pregnant women because it can be increased under the influence of estrogenic hormones [15]. However, any increase in the levels of aminotransferases, bilirubin, alkaline phosphatase, γ-glutamyl-transpeptidase, and 5′-nucleotidase above the normal unchanged limits in pregnancy should be an indicator of liver or biliary disease that needs immediate investigation [16]. In our patient, who was in the third trimester of pregnancy, the increase in bilirubin, transaminases, and alkaline phosphatase was borderline. Additionally, the mild leukocytosis and the increase in C-reactive protein could be attributed to pregnancy and not to the presence of an intra-abdominal infection. In our case, the diagnosis of acute cholecystitis was based on ultrasound examination.

Ultrasound is the imaging modality of choice for the diagnosis of acute cholecystitis in pregnant women. It is a rapid, non-invasive method, with an accuracy of detecting gallstones in 95%-98% of cases. Ultrasound findings such as the presence of gallstones in the gallbladder, thickening of the bladder wall (>3mm) with a layered appearance, pericholecystic fluid collection, and dilation of the common bile duct are indicative of acute inflammation of the gallbladder wall [17]. Moreover, unlike computed tomography, ultrasound does not require the use of ionizing radiation, which is best avoided in pregnancy as it is a known teratogen [18]. Additionally, magnetic resonance imaging is now considered a fairly safe imaging modality for diagnosing abdominal pain of different etiologies at any stage of pregnancy and has completely replaced the potentially "damaging" for the fetus computed tomography scan. Magnetic resonance imaging findings, combined with clinical and ultrasound findings, are thought to significantly aid in the evaluation of pregnant women with abdominal pain [19]. Additionally, the contribution of magnetic resonance imaging in performing diagnostic procedures, such as magnetic resonance cholangiopancreatography, is well established and plays a crucial role in the comprehensive evaluation and further treatment of the patient [20]. In our case, the clinical findings were not typical of acute cholecystitis. Performing computed tomography was not recommended due to the use of ionizing radiation. Emergency magnetic resonance imaging was not available in our hospital. Our recommendation to perform a scheduled magnetic resonance imaging was not accepted by the patient. The diagnostic investigation and subsequent treatment of the pregnant woman were based mainly on the ultrasound findings.

The management of acute cholecystitis in pregnancy is similar to that of non-pregnant patients and is based on conservative supportive antibiotic therapy and open or laparoscopic cholecystectomy. Conservative medication is often better accepted by pregnant women due to fears for fetal health and concerns about the outcome of pregnancy compared to surgical treatment [21,22]. Similarly, in our case, our patient's preference after the diagnosis of acute cholecystitis was to avoid surgery and to receive medication, despite her fears about the risk of teratogenesis from the administered drugs. The basic principles of conservative management include nil per os careful intravenous fluid administration, and parenteral administration of pain medication and antibiotics. First-line treatment consists of amoxicillin, ampicillin, or second-generation cephalosporins in combination with metronidazole, depending on the severity of the disease [23]. Treatment of cholelithiasis with litholytic drugs such as ursodeoxycholic acid, chenodeoxycholic acid, and ezetimibe should be carefully considered in pregnant women, although in isolated cases where these drugs were administered, no maternal or fetal side effects were observed [24].

Despite the benefits of conservative treatment and its wide acceptance by the majority of patients, Barut et al., analyzing the results of their study, showed that in 27%-36% of cases, symptoms do not fully resolve after conservative treatment, resulting in prolonged abdominal pain and vomiting, causing intrauterine fetal distress and uterine contractions with an increased risk of miscarriage and preterm labor [25]. Therefore, the major dilemma in the management of cholecystitis in pregnancy remains the timely and correct decision of surgical intervention. In a recent study (2023), Zhang et al. argue that in patients with acute cholecystitis in the last trimester of pregnancy, as in our patient, the appropriate therapeutic approach should be applied after careful evaluation of the severity of disease symptoms and the willingness of pregnant patients. It is appreciated that in uncomplicated conditions with mild symptoms, it is appropriate that conservative drug therapy be followed and any invasive procedure be postponed until after delivery [26]. In patients with moderate or severe symptoms, percutaneous transhepatic drainage of the gallbladder is initially indicated to decrease the severity of symptoms and allow for the possibility of performing a postpartum cholecystectomy. Immediate cholecystectomy in the antenatal period by open or laparoscopic approach should be performed in cases of inadequate symptom control after conservative treatment [26]. Recently, in 2024, Hantouli et al. showed that the decision to perform cholecystectomy during pregnancy, especially in the last trimester, may be an opportunity to improve perinatal outcomes in pregnant women with acute cholecystitis [27]. Laparoscopic cholecystectomy during pregnancy appears to have lower morbidity compared to open cholecystectomy [28].

The prognosis of acute cholecystitis in pregnant women depends on the gestational age and delay in diagnosis. Delayed diagnosis may lead to serious complications such as acute pancreatitis, cholangitis, gallbladder fistula formation, gallbladder perforation, and the development of generalized peritonitis and septicemia, with significantly increased rates of maternal and perinatal morbidity and mortality [28,29]. Additionally, fetal loss in early pregnancy, premature uterine contractions, and the onset of preterm labor are common complications of acute cholecystitis in pregnant women [21].

## Conclusions

Acute cholecystitis is the second most common non-obstetric cause of exploratory laparotomy in pregnancy after acute appendicitis. A multidisciplinary approach is essential in pregnant women, particularly when clinical manifestations are atypical. Skill in decision-making by an experienced team of surgeons is of primary importance to adequately evaluate the possibility of surgical intervention during pregnancy.
